# The peptidoglycan and biofilm matrix of *Staphylococcus epidermidis* undergo structural changes when exposed to human platelets

**DOI:** 10.1371/journal.pone.0211132

**Published:** 2019-01-25

**Authors:** Maria Loza-Correa, Juan A. Ayala, Iris Perelman, Keith Hubbard, Miloslav Kalab, Qi-Long Yi, Mariam Taha, Miguel A. de Pedro, Sandra Ramirez-Arcos

**Affiliations:** 1 Centre for Innovation, Canadian Blood Services, Ottawa, Canada; 2 Department of Biochemistry, Microbiology and Immunology, University of Ottawa, Ottawa, Canada; 3 Centro de Biología Molecular Severo Ochoa, Universidad Autónoma de Madrid, Madrid, Spain; 4 Agriculture and Agri-food Canada, Ottawa, Ontario, Canada; National Institute of Technology Rourkela, INDIA

## Abstract

*Staphylococcus epidermidis* is a bacterium frequently isolated from contaminated platelet concentrates (PCs), a blood product used to treat bleeding disorders in transfusion patients. PCs offer an accidental niche for colonization of *S*. *epidermidis* by forming biofilms and thus avoiding clearance by immune factors present in this milieu. Using biochemical and microscopy techniques, we investigated the structural changes of the peptidoglycan (PG) and the biofilm matrix of *S*. *epidermidis* biofilms formed in whole-blood derived PCs compared to biofilms grown in glucose-supplemented trypticase soy broth (TSBg). Both, the PG and the biofilm matrix are primary mechanisms of defense against environmental stress. Here we show that in PCs, the *S*. *epidermidis* biofilm matrix is mainly of a proteinaceous nature with extracellular DNA, in contrast to the predominant polysaccharide nature of the biofilm matrix formed in TSBg cultures. PG profile studies demonstrated that the PG of biofilm cells remodels during PC storage displaying fewer muropeptides variants than those observed in TSBg. The PG muropeptides contain two chemical modifications (amidation and O-acetylation) previously associated with resistance to antimicrobial agents by other staphylococci. Our study highlights two key structural features of *S*. *epidermidis* that are remodeled when exposed to human platelets and could be used as targets to reduce septic transfusions events.

## Introduction

*Staphylococcus epidermidis* is part of the normal human skin microbiome [1, 2]. It helps in the maintenance of a healthy skin, outcompeting harmful microorganisms such as *Staphylococcus aureus* [3, 4]. Although *S*. *epidermidis* does not produce virulence factor such as exotoxins, it has emerged as a significant opportunistic pathogen associated with healthcare-associated infections [3, 5]. Its ability to adhere to plastics for medical use and subsequent formation of surface-attached aggregates of bacteria known as biofilms is its most important virulence trait [3–5]. In transfusion medicine, *S*. *epidermidis* is the most frequent aerobic pathogen isolated from contaminated platelet concentrates (PCs), a blood product administered to patients with bleeding disorders [6]. PCs can be manufactured by pooling buffy coat fractions obtained from whole blood donations of four to five donors. The pooled buffy coats are suspended in the plasma of one of the donors. Alternative, PCs can be collected from a single donor using an apheresis (centrifugation) device. Independently of the manufacturing method, the final PC product can be suspended in plasma or in a mix of plasma and a buffering additive solution [7]. PCs are susceptible to bacterial contamination due to their storage conditions in gas-permeable plastic bags, containing high concentration of glucose, incubated at 20–24°C with agitation for up to 7 days, all of which are amenable for bacterial growth. A number of practices such as donor skin disinfection, diversion of the first aliquot of the donated blood, PC sterility testing using culture methods, and pathogen reduction technologies, have been implemented to minimize the risk of transfusing bacterially–contaminated blood products [7]. Biofilm formation in PCs has been shown to increase missed detection during sterility screening [8, 9]. Proliferation of *S*. *epidermidis* in the PC storage environment may be linked to its ability to form biofilms, which confer protection from host defense molecules such as antimicrobial peptides (AMPs) derived from platelets [2, 10–14]. We have recently demonstrated that although AMPs could prevent biofilm formation by *S*. *epidermidis*, established mature staphylococcal biofilms are resistant to the bactericidal action of these immune factors [15]. Interestingly, *S*. *epidermidis* isolates displaying a biofilm-negative phenotype convert to a biofilm-positive phenotype when grown in PCs, dependant on the presence of plasma factors [8, 16, 17]. Typical biofilm formation in *S*. *epidermidis* depends on the production of the exo-polysaccharide intracellular adhesin (PIA), encoded by the *icaADBC* operon. PIA mediates the accumulation stage of biofilm formation and is the main component of the biofilm matrix [18, 19]. Some PIA-negative strains are able to form biofilms in an *ica*-independent manner involving several cell wall anchored proteins [18, 20], while others display a biofilm-negative phenotype [21]. Interestingly, certain strains are able to switch between PIA-dependent and protein-dependent biofilm formation with chemical changes in the structure of the biofilm matrix [22, 23]. Biofilm formation is a highly regulated process that involves a wide number of molecular factors including cell-surface proteins involved in adhesion to biotic or abiotic surfaces (Bhp, AltE, SSP-1 and SSP2) or the biofilm accumulation phase (Aap, Embp); extracellular DNA (eDNA); and teichoic acids (TA) [24, 25]. Importantly, relative amounts of the biofilm matrix components such as PIA and TA are dependent on growth conditions [26]. The matrix is a mesh of molecules that interact with each other, for example, Aap can be covalently linked to peptidoglycan (PG) forming fibril-like structures [27, 28]. Similarly, TA increase bacterial adhesion to fibronectin-coated surfaces and contribute to biofilm formation [24].The study of the PG profile in *S*. *epidermidis* has not gained much attention in comparison to other staphylococci [29, 30]. Here we show that changes in the PG and biofilm matrix structure occur during biofilm formation of *S*. *epidermidis* in buffy coat PCs suspended in plasma. We explored the PG composition of *S*. *epidermidis* cells from mature biofilms formed in PCs compared to biofilm cells grown in laboratory media under optimal growth conditions. Furthermore, we explored the changes in the biofilm matrix composition and structure of *S*. *epidermidis* biofilms formed in PCs.

## Materials and methods

### Bacterial strains and culture methods

Four *S*. *epidermidis* isolates from the strain collection of Canadian Blood Services were used in this study, including two isolates form contaminated PCs ST10002 (biofilm-positive) and ST11003 (biofilm-negative), and two isolates from the skin of healthy volunteers, AZ22 and AZ39 (both biofilm-positive) [31]. *S*. *epidermidis* 9142 (biofilm-positive) and 9142Δ*icaA* (biofilm-negative) [32] were used as biofilm-positive and biofilm-negative controls, respectively. Bacterial cultures were routinely grown under standard conditions in Trypticase Soy Broth (TSB) at 37°C or in PCs under standard PC storage conditions (20–24°C, under constant agitation for five days).

### Congo red agar assay

Production of PIA was tested as described previously [33]. Congo red agar plates were prepared with sterile Brain Heart Infusion agar with the addition of filter-sterilized 0.8 g/ L Congo red stain (Sigma-Aldrich Canada Ltd., Oakville, Canada) and 36 g/ L of saccharose (Sigma-Aldrich Canada Ltd., Oakville, Canada). Qualitative PIA production by *S*. *epidermidis* isolates were tested by plating on Congo red agar plates followed by incubation for 48 h at 37°C. PIA-producing isolates form black, dry, crusty colonies due to the interaction of the Congo red dye with the polysaccharide matrix, while PIA-negative isolates form pink or reddish creamy colonies. *S*. *epidermidis* 9142 and *S*. *epidermidis* 9142 Δ*ica*A were used as positive and negative controls for PIA production, respectively.

### Platelet concentrates preparation

Ethical approval for this study was granted by the Canadian Blood Services Research Ethical Board. PCs were prepared from whole blood donations using the buffy coat method at the Canadian Blood Services Network Centre for Applied Development (netCAD, Vancouver, BC, Canada) in agreement with Canadian Blood Services procedures [34]. PCs were suspended in plasma and shipped to the Canadian Blood Services Centre for Innovation microbiology laboratory in Ottawa, Ontario, Canada, where they were screened for baseline bacterial contamination following standard procedures [35]. Buffy coat pooled PCs are required to contain ≥ 240 x 10^9^ platelets/pool.

### Biofilm matrix disruption assay

Biofilm formation assays were performed as described [16] briefly: overnight cultures were adjusted to ~10^7^ colony forming units (CFU)/ml, 3 ml aliquots of bacterial suspension in TSBg or PCs were added to 6-well polystyrene plates (Falcon, Corning Inc., Durham, NC). The 6-well plates were incubated for 24 hours at 37°C to allow for biofilm formation in TSBg or for 5 days under PC storage conditions for biofilm formation in PCs. Following incubation, supernatants were discarded, and biofilms were washed three times using PBS (pH 7.4). To semi-quantify the protein composition of biofilm matrices, 2.9 ml of 100 μg/ml proteinase K (Sigma-Aldrich, Oakville, Canada) was added to the grown biofilms in a 20mM Tris and 100 mM NaCl buffer (pH = 7.5) for 2h as previously described [36]. Alternatively, to determine the DNA composition of the biofilm matrices, 2.9 ml of 0.5 mg/ml DNase I (DN25; Sigma) in 5 mM MgCl_2_ was added to grown biofilms for 24h. Following incubation with the respective disruptive agent (proteinase K or DNaseI), residual biofilms were quantified using the semi-quantitative crystal violet assay [37]. Biofilms were rinsed three times with 1X PBS and stained with 0.3% Gram crystal violet dye (BD Biosciences, MD, USA) for 30 min. Staining was eluted with 80:20 ethanol: acetone solution for 15 min. Six 200 μL aliquots of the de-stained solution from each well were transferred to a 96-well polystyrene plate (Falcon, Corning Inc., Durham, NC), and the absorbance at 492 nm was measured with an Expert Plus microplate reader (Biotech, Montreal, Canada). To quantify and determine formation of biofilm, we followed the recommendations for assessment of biofilm production by staphylococci [38]. For assays in PCs, the only modifications to the above protocol were that 6 ml of disrupting agent was added to each well in order to cover the biofilm formed on the sides of the well.

### Detection of the PIA by immune dot-blot test

Immuno-dot blots were performed to detect PIA production by *S*. *epidermidis* isolates. Briefly, *S*. *epidermidis* biofilms were grown in TSBg and PCs. Supernatants were discarded, and the adhered biofilms were scraped off the plates and suspended in 1X PBS (pH = 7.4) followed by centrifugation at 5000g for 10 min and boiling in 0.5 M EDTA (pH = 8.0; Thermo Fisher Scientific, Whitby, Canada) for 5min to lyse cells and release any cell wall-bound PIA into the supernatants. The boiled samples were centrifuged, and the supernatants were treated with 10 μL of 10 mg/ml proteinase K (Sigma-Aldrich, Oakville, Canada) for 1 h at 37°C. Proteinase K activity was stopped by heating at 95 °C for 5 min. A 2 μL sample of each supernatant was spotted onto a nitrocellulose membrane. The membrane was blocked using bovine serum albumin and then incubated with 1:5,000 anti-*S*. *epidermidis* PIA-specific antiserum kindly provided by Dr. D. Mack (Swansea University, UK) [39]. After washing, the bound antibodies were detected with an alkaline-phosphatase conjugated anti-rabbit IgG (Sigma-Aldrich, Oakville, Canada). The membrane was washed, and subsequently incubated with 5-bromo-4-chloro-3'-indolyphosphate and nitro-blue tetrazolium developer (Sigma-Aldrich, Oakville, Canada) for 5 min. Membranes were visualized using a MultiImage Light Cabinet and AlphaImager 2200 software (Fisher Scientific, St-Laurent, Quebec). *S*. *epidermidis* 9142 was used as positive control for PIA production, while *S*. *epidermidis* 9142 Δ*ica*A was used as the negative control.

### Statistical analysis

Mean and standard deviation were calculated for each strain in both growth environments (TSBg and PCs). A mixed model analysis was performed to test the difference between treated and untreated biofilms with proteinase K or DNase I. In the model, replication ID was fitted as random effects to control the potential clustering effect. Data from the two study environments were analysed separately. A p-value of <0.05 was considered statistically significant. All the analyses were performed in SAS software (SAS Institute Inc., SAS/STAT 9.3).

### Fluorescence Microscopy of *S*. *epidermidis* biofilms in TSBg and PCs

The composition of *S*. *epidermidis* biofilms was observed by Confocal Laser Scanning Microscopy (CLSM). Biofilms of *S*. *epidermidis* strains ST10002 and AZ39 were grown in either TSBg or PCs on PC-bag coupons (~7x10 mm), cut from Terumo 804440 bags (TerumoBCT, Lakewood, CO); the coupons were placed in 6-well polystyrene plates (Falcon, Corning Inc., Durham, NC). Initial cultures were inoculated with 10^7^ CFU/ml. The coupons were rinsed with 1X PBS (pH = 7.4), dried briefly in a Petri dish and frozen at -20°C or kept in PBS at 4°C. Biofilms were then labelled with three fluorescent dyes as follows: (I) Wheat germ agglutinin (WGA) conjugated with Oregon Green-488 (Thermo Fisher Scientific, Waltham, MA, USA) (WGA-OG488), which labels polysaccharides by binding to N-acetylglucosamine and sialic acid residues, 5 μm in dH_2_O/15 min. (II) Film tracer SYPRO Ruby Biofilm Matrix Stain (Thermo Fisher Scientific, Waltham, MA, USA), which labels most types of proteins, for 15 min prior to live confocal imaging (III) Coupons were then mounted on slides under coverslips in DAPI-Fluoromount-G (Electron Microscopy Sciences, Hatfield, PA, USA), containing 4’,6-diamidino-2-phenylindole (DAPI), which labels DNA. Coupons were rinsed for 1 min in ultrapure water following each of the first two staining steps.

Samples were imaged using a Zeiss LSM800 Airyscan CLSM (Carl Zeiss MicroImaging, Göttingen, Germany). For visualisation of WGA, SYPRO and DAPI staining, excitation lasers of 488 nm, 405 nm and 405 nm were used respectively. For the TSBg samples, emission bands of 505–555 nm for WGA, 555–700 nm for SYPRO, and 410–505 nm for DAPI were acquired using the GaAsP detectors and a Plan-Apochromat 40x NA 1.4 oil objective. For the PC samples, emission bands of 485–560 nm for WGA, 554–700 nm for SYPRO, and 400–530 nm for DAPI were acquired using the high resolution Airyscan detector and a Plan-Apochromat 63x NA 1.4 oil objective. Images were processed with ZEN 2.3 software (Carl Zeiss MicroImaging, Göttingen, Germany) and Adobe Photoshop CS6 (http://www.adobe.com/).

For the visualization of eDNA in the biofilm matrix and the DNA in the bacterial cells, a combination of TOTO-1 (Thermo Fisher Scientific, Waltham, MA, USA) with the cell permeable SYTO 60 (Thermo Fisher Scientific, Waltham, MA, USA) [40] was used on biofilms samples grown in PCs. CLSM analysis of the eDNA content in the biofilm matrix of *S*. *epidermidis* ST10002 (PIA^+^) and AZ39 (PIA^-^) in PC was performed using a specific dye for eDNA, SYTO-60. Biofilms were stained with the eDNA specific cell-impermeant nucleic acid stain TOTO-1 (2 μm for 2 min) and counterstained with the cell-permeant nucleic acid stain SYTO-60 (10 μm for 2 min). Samples were visualized using excitation lasers of 488 nm and 640 nm for TOTO-1 and SYTO-60 respectively. An emission band of 450–630 nm was acquired for TOTO-1 using the Airyscan detector and an emission band of 656–700 nm was acquired for SYTO-60 using the GaAsP detector. Differential interference contrast microscopy (DIC) was used to observe the biofilms without stain. The eDNA staining patterns observed for ST10002 (PIA^+^) and AZ39 (PIA^-^) were similar. Biofilms were observed with a 40x objective. Scale bar = 5 μm.

### Scanning electron microscopy (SEM)

Biofilms of *S*. *epidermidis* strains ST10002 and AZ39 were grown in either TSBg or PCs on PC-bag coupons (~7x10 mm), from Terumo 804440 bags (TerumoBCT, Lakewood, CO), which were placed in 6-well polystyrene plates (Falcon, Corning Inc., Durham, NC). Initial cultures were inoculated with 10^7^ CFU/ml. The coupons were rinsed with 1X phosphate buffer saline (pH = 7.4). For SEM imaging, the aggregates were fixed in a 2.5% v/v glutaraldehyde in a 0.1 M sodium cacodylate buffer (pH 7.0) for 10 min at room temperature (22–24 °C) and dehydrated with graded ethanol concentrations (20, 50, 70, and 100%). The coupons were then critical point dried, mounted an aluminum SEM stubs, coated with a 9 nm thin layer of gold and examined in a Philips XL-30 ESEM scanning electron microscope operated at 7.5 kV and a 7.5 mm working distance. Images (2576x1936 pixels) were obtained in TIFF format.

### Peptidoglycan extraction

Peptidoglycan was prepared from *S*. *epidermidis* biofilm cultures grown in TSBg or PCs following a previously described protocol [41] with slight modifications. Briefly, 50 ml bacterial cultures adjusted to of 10^7^−10^8^ CFU/ml prepared in in culture flasks (Falcon, Corning Inc., Durham, NC) were incubated to allow for biofilm formation for 24h/37°C in TSBg, or for 5 days/22°C with agitation in PCs. After incubation, supernatant was discarded and biofilms were rinsed with 1X PBS (pH = 7.4). Adhered biofilms were then scraped off the flask and suspended in 10 ml 1X PBS (pH = 7.4). Cells were harvested by centrifugation for 15 min at 4000x g at room temperature, resuspended in 3 ml of ddH_2_O and added to a boiling 10%(v/v) SDS solution vigorously stirred. The sample was boiled overnight in a water bath without stirring. Sacculi was concentrated by centrifugation for 15 min and the pellet was washed until no SDS was detected. The pellet, free of SDS, was suspended in 0.5 ml of 10 mM Tris-HCl, 0.06% NaCl (pH 7.2) and digested with α-amylase (100 μg/ml) for 90 min at 37°C and then with 100 μg/ml pronase-E for 1h at 60°C. PG was further digested in 50 mM phosphate buffer (pH 4.9) with 20 μg/ml Cellosyl (Hoechst AG, Frankfurt, Germany) at 37°C overnight. The insoluble material was removed by centrifugation, and soluble muropeptides were reduced with sodium borohydride and frozen at -70°C.

### Analysis of muropeptides using high performance liquid chromatography (HPLC) and MALDI-TOF mass spectrometry

Muramidase-digested samples were analyzed by high-performance liquid chromatography (HPLC) as previously described [42]. Briefly, samples were filtered and injected into the HPLC. Separations were performed on a Waters Breeze 2 HPLC System (equipped with Waters 1525 Binary HPLC Pump and Waters 2489 UV/Visible Detector). Separation of individual muropeptides was performed in a linear gradient, the column was equilibrated at 45°C, and eluted compounds were detected at a wavelength of 204 nm. Individual muropeptides resolved by HPLC, were analyzed by MALDI-TOF carried out at the CMBSO Protein Chemistry Facility at the Universidad Autonoma de Madrid, Spain.

### Relative quantification of the muropeptides

The relative quantification of the muropeptide content was performed calculating the normalized percentage of the relative corresponding area under each peak, from which the chemical composition was determined (peaks 1 to 23) adjusted to 100% in each PG profile.

## Results

### The biofilm matrix of *S*. *epiderm*idis formed in PCs displays high protein content

**Fig 1:**
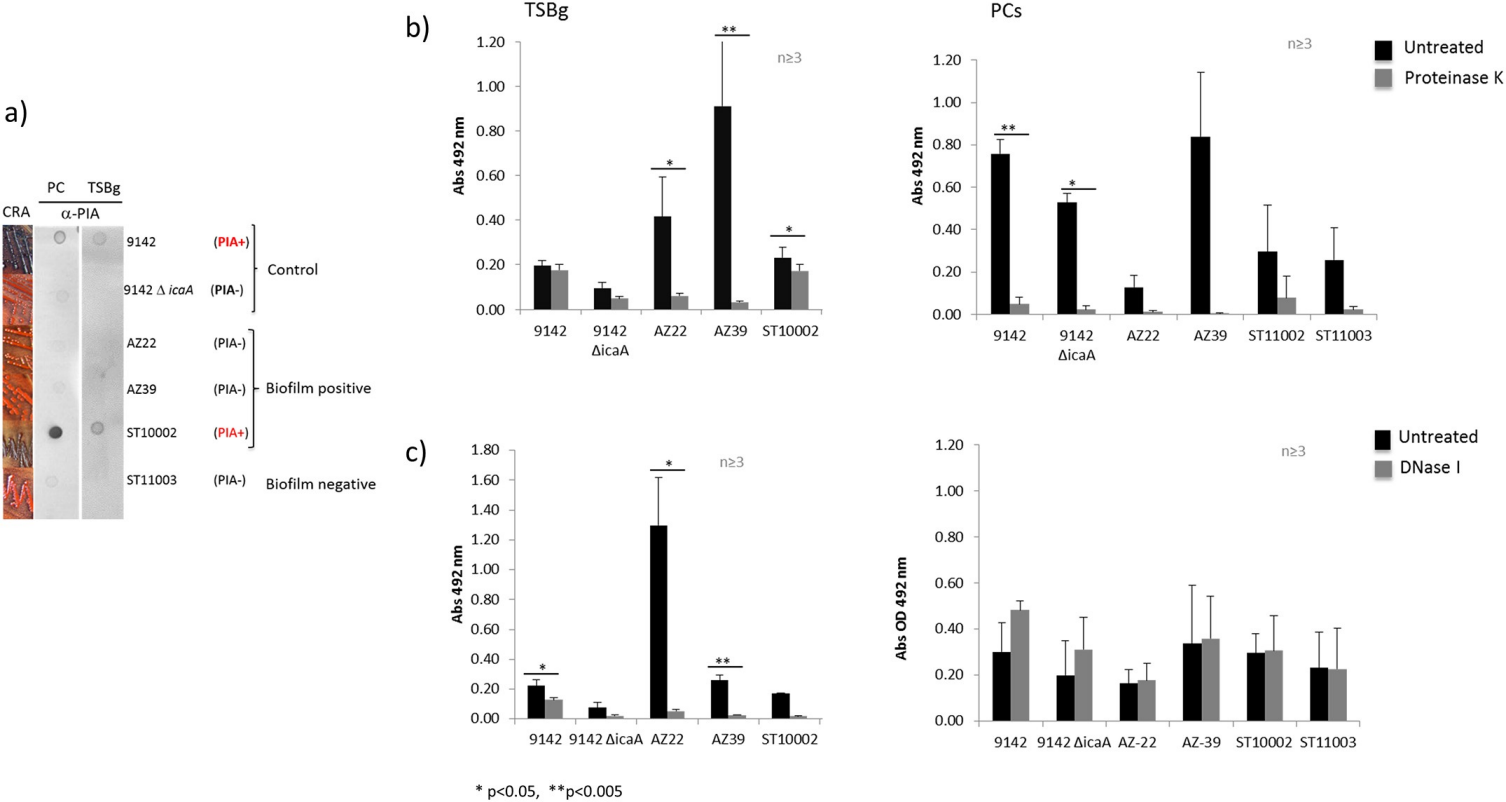
Biofilm matrix composition of *S*. *epidermidis*. a) PIA production by *S*. *epidermidis* biofilms. Left panel, cultures on Congo red agar plates: PIA production is shown by formation of dark crusty colonies while PIA-negative strains form smooth pink colonies. Dot-blots using anti-PIA antibodies shows dark circles for PIA^+^ strains. b) Protein detection in *S*. *epidermidis* biofilms. A proteinase K disruption assay show presence of a proteinaceous matrix in *S*. *epidermidis* biofilms grown in TSBg or PCs. Black bars show untreated biofilms while grey bars show disrupted biofilms. c) eDNA detection in *S*. *epidermidis* biofilms. A DNase I disruption assay show presence of eDNA in the matrix of *S*. *epidermidis* biofilms grown in TSBg. None of the strains exhibited biofilm disruption with DNase I treatment when grown in PCs.. Black bars show untreated biofilms while grey bars show treated biofilms. * (p < 0.05) and ** (p < 0.005).

Four *S*. *epidermidis* isolates (ST10002, ST10003, AZ22, and AZ39) were grown as biofilms in PCs and in glucose-supplemented Trypticase Soy Broth (TSBg). ST10002, AZ22 and AZ39 are biofilm-positive in both TSBg and PCs while *S*. *epidermidis* ST11003 displays a biofilm-negative phenotype in TSBg [31] but converts to biofilm-positive in PCs. Production of a PIA-based biofilm matrix was assayed by immune dot blot using *S*. *epidermidis* 9142 and 9142Δ*icaA* [32] as PIA-positive (PIA^+^) and PIA–negative (PIA^-^) controls, respectively. Fig 1a shows that a PIA-based biofilm matrix was detected in ST10002 grown in PCs and TSBg, but it was absent in AZ39 and AZ22. A Congo red agar assay supported these results showing positive PIA production by *S*. *epidermidis* ST10002 and a PIA-negative phenotype for strains AZ39, AZ22 and ST11003 (Fig 1a).

Mature biofilms of the four isolates were treated with proteinase K to determine the relative amounts of protein in the biofilm matrix [36]. In TSBg, PIA^+^ *S*. *epidermidis* 9142 showed approximately 10% of biofilm reduction after proteinase K treatment (p = 0.1206) while biofilms of *S*. *epidermidis* ST10002, also a PIA^+^ strain, were reduced by 25.4% post proteinase K treatment (p = 0.0301) (Fig 1b and S1 Table). In contrast, strains AZ22 and AZ39, which classify as moderate and strong biofilm formers, respectively, according to the classification of Stepanovic and colleagues [38], and do not carry the *icaA* and *icaD* genes (i.e., PIA^-^) [31], were highly susceptible to proteinase K treatment. After exposure to proteinase K, *S*. *epidermidis* AZ22 biofilms showed 85.6% reduction (p = 0.0229) and AZ39 biofilms displayed a 96.4% reduction (p = 0.0044) (Fig 1b and S1 Table). Results showed that the chemical composition of the matrix of *S*. *epidermidis* biofilms formed in PCs by all strains was predominantly of a proteinaceous nature. Biofilms were highly disrupted (>73%) after treatment with Proteinase K (Fig 1b).

To determine the relative amounts of eDNA in the biofilm matrix, mature biofilms were treated with DNase I. In TSBg, biofilms of strains 9142, AZ22, AZ39 and ST10002 were significantly disrupted after incubation with DNase I for 24h (Fig 1c and S2 Table). Percent biofilm reduction for strains 9142, AZ22, AZ39 and ST10002 was 42.2% (p = 0.0219), 95.9% (p = 0.0206), 90.0% (p = 0.0077) and 88.8% (p = 0.0004), respectively (S2 Table). None of the strains exhibited biofilm disruption with DNase I treatment when grown in PCs. This was likely due to the fact that *S*. *epidermis* biofilms incubation with DNase I for several days could be less susceptible to disruption [43] and/or an excess of eDNA in the growing medium that can prevent the effect of DNase I treatment as shown in *S*. *aureus* [44]. To overcome these issues, we further investigated the presence of eDNA in the *S*. *epidermidis* biofilms formed in PCs, using microscopy techniques.

### Structure of the *S*. *epidermidis* biofilm matrix is remodelled in PCs compared to laboratory optimal conditions and it is mainly made of proteins and eDNA

We investigated the macromolecular biofilm matrix composition and the biofilm structure of two *S*. *epidermidis* strains ST10002 (PIA^+^) and AZ39 (PIA^-^) in PCs and TSBg. Biofilms were grown on plastic coupons prepared from PC bags. The biofilm matrices of both strains were simultaneously stained with fluorescent dyes for polysaccharides (WGA-OG488), proteins (SYPRO-Ruby), and DNA (DAPI) and analyzed using Confocal Laser Scanning Microscopy (CLSM). Scanning electron microscopy (SEM) was used to explore the structure of the biofilms on the coupons.

**Fig 2:**
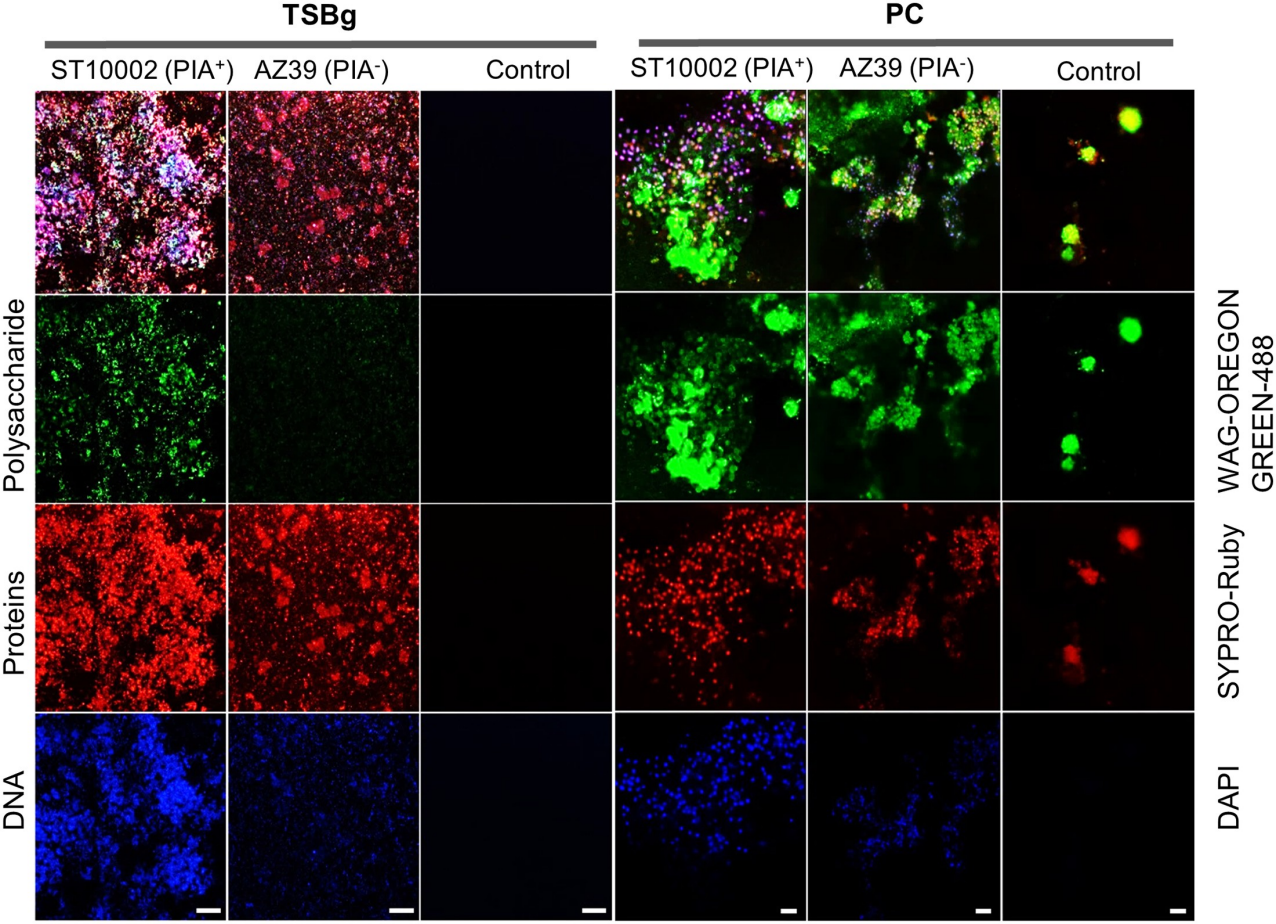
Macromolecular composition of the *S*. *epidermidis* biofilm matrix. CLSM analysis of the biofilm matrix composition of *S*. *epidermidis* ST10002 (PIA^+^) and AZ39 (PIA^-^). WGA-OG488 staining shows polysaccharide content attributed to PIA production. SYPRO-Ruby staining shows proteins contained in the biofilm matrix and DAPI staining was used to detect DNA and visualize bacteria. TSBg biofilms were observed with a 40x objective. Scale bar = 20 μm. PC biofilms were observed with a 63x objective. Scale bar = 5 μm. TSBg coupons were stored at 4°C in PBS 1x before staining while PC coupons were rinsed with PBS 1X, dried, and stored at -20°C before staining.

In TSBg, ST10002 displayed extensive staining by both WGA-OG488 and SYPRO-Ruby, while AZ39 showed staining by SYPRO-Ruby but little by WGA-OG488 (Fig 2). This suggests that in TSBg, the biofilm matrix of ST10002 comprises both polysaccharides and proteins while the biofilm matrix of AZ39 is predominantly proteinaceous. In both strains, the WGA-OG488 and SYPRO-Ruby staining overlapped the DAPI-stained bacteria and eDNA (Fig 2).

Coupons incubated in TSBg or PCs without bacteria were used as controls. The TSBg control coupons did not show any staining (Fig 2). The PC control coupons, however, showed both WGA-OG488 and SYPRO-Ruby staining of globular structures, that did not show any DAPI staining, indicative of PC polysaccharide and protein components adsorbed by the plastic coupons (Fig 2). In PCs, ST10002 and AZ39 were observed to associate with these adsorbed PC components, perhaps using them as foundations from which to further develop biofilms (Fig 2). Whereas WGA-OG488 staining in PC grown ST10002 and AZ39 seemed to be primarily associated with PC components, SYPRO-Ruby staining more closely overlapped the DAPI-stained bacteria (Fig 2). Thus, the biofilm matrix of both ST10002 and AZ39 biofilms grown in PCs is associated with adsorbed protein and polysaccharide PC components but is itself more proteinaceous.

**Fig 3:**
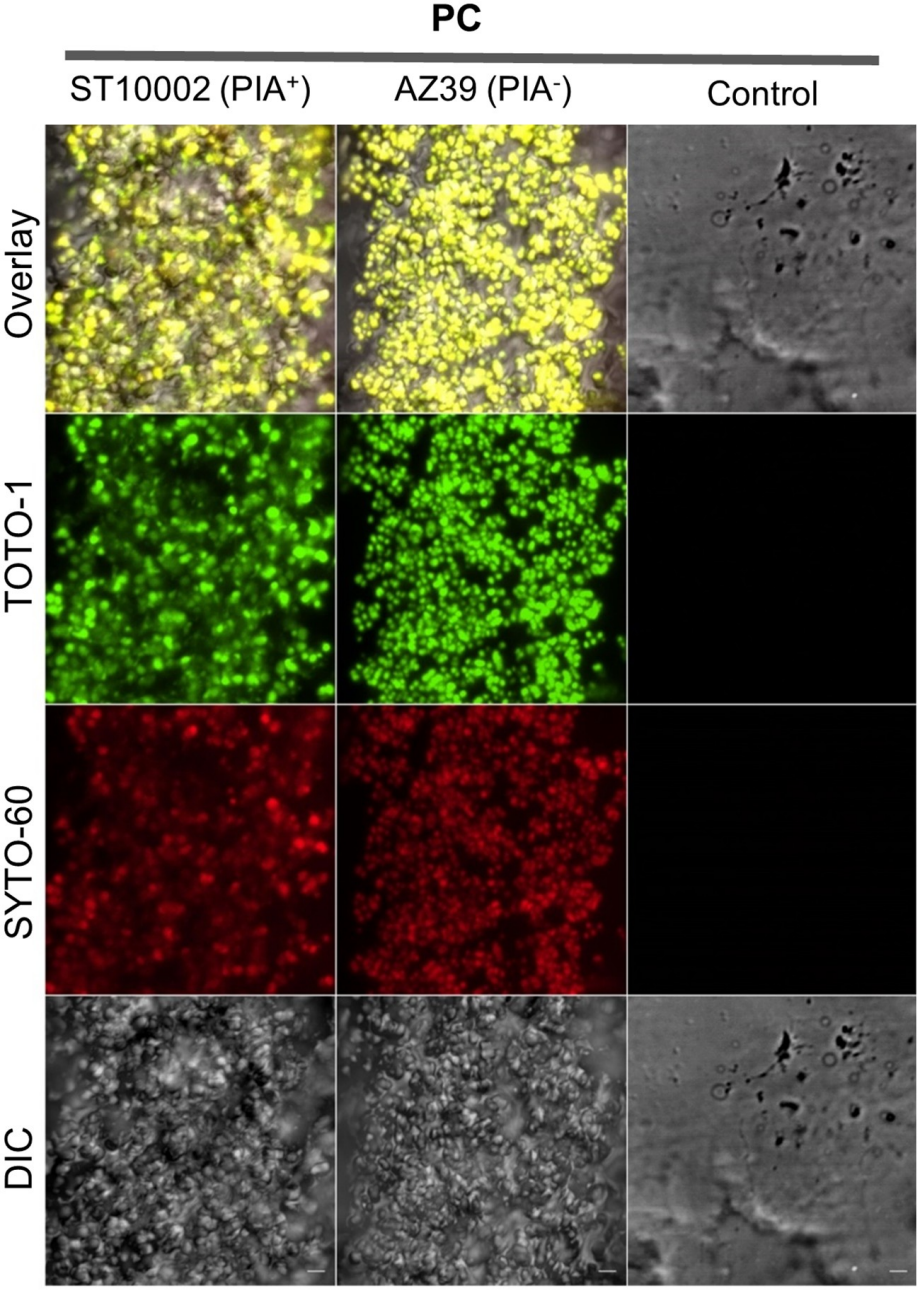
eDNA presence in *S*. *epidermidis* biofilms formed in PCs. CLSM analysis of the DNA content of the biofilm matrix of *S*. *epidermidis* ST10002 (PIA^+^) and AZ39 (PIA^-^) formed in PCs. Biofilms were stained with the eDNA specific cell-impermeant nucleic acid stain TOTO-1 and counterstained with the cell-permeant nucleic acid stain SYTO-60. Samples were visualized using excitation lasers of 488 nm and 640 nm for TOTO-1 and SYTO-60 respectively. An emission band of 450–630 nm was acquired for TOTO-1 using the Airyscan detector and an emission band of 656–700 nm was acquired for SYTO-60 using the GaAsP dectector. Differential interference contrast microscopy (DIC) was used to observe the biofilms without stain. The eDNA staining patterns observed for ST10002 (PIA^+^) and AZ39 (PIA^-^) were similar. Biofilms were observed with a 40x objective. Scale bar = 5 μm.

Since DAPI stained both the bacterial DNA and biofilm eDNA, precluding conclusions regarding differential eDNA content between the two strains, the fluorescent eDNA stain TOTO-1 and the total DNA counterstain SYTO-60 were used to examine the eDNA content of the biofilms. In PCs, both *S*. *epidermidis* strains showed similar staining patterns (Fig 3).

**Fig 4:**
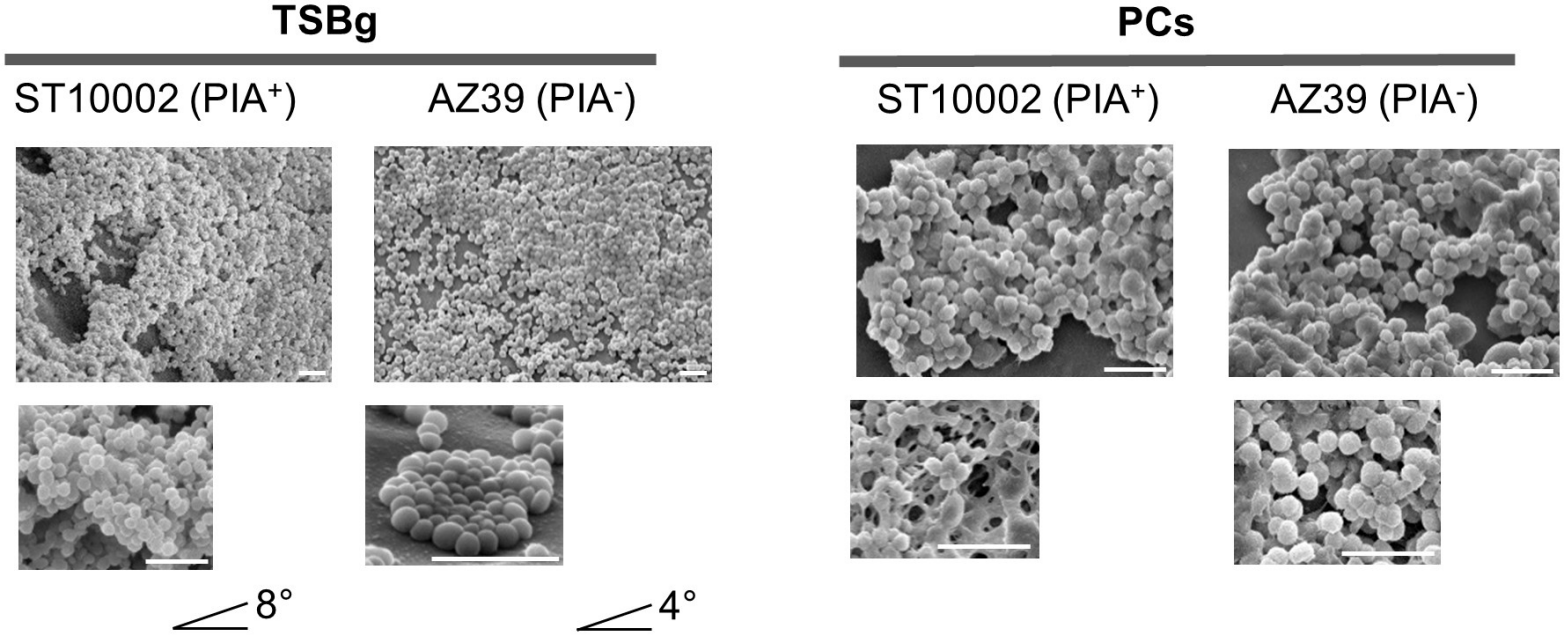
Biofilm architecture of *S*. *epidermidis* biofilms. SEM micrographs showed different biofilm structures of ST10002 and AZ39 in PCs compared to TSBg. In TSBg, the samples were tilted < 8° and < 4°, respectively to allow for visualization of thickness of the biofilms. While ST10002 formed thick multilayered biofilms, AZ39 formed monolayered biofilms. By contrast, in PCs, both strains displayed a thick multilayered biofilm. Scale bar = 5 μm.

SEM micrographs of the biofilms of ST10002 and AZ39 formed in PCs compared to TSBg revealed a different structure. In TSBg, ST10002 formed thick multilayered biofilms while AZ39 formed monolayered biofilms (Fig 4). In PCs, the structure of the biofilms of ST10002 and AZ39 were not visually different and biofilms from both strains displayed a thick multilayered biofilm structure (Fig 4).

#### The PC milieu induces structural changes in the peptidoglycan of *S*. *epidermidis* biofilm cells

Because structural changes in the biofilm matrix may also affect the composition of the cell wall of biofilm cells, we studied the composition of the PG of *S*. *epidermidis* ST10002 and AZ39 biofilms cells grown in TSBg and PCs using HPLC and MS techniques. The analysis was restricted to early eluting components (muropeptides), due the complexity of the staphylococcal peptidoglycan. The muropeptides, upon MS examination, were found to represent variations of the basic monomeric subunit N-acetylglucosamine (NAG)-N-acetylmuramic acid (NAM)-L-Ala-D-Gln (γ-L-Lys-[ε-Gly_5_]-D-Ala-D-Ala, similar to the basic monomers of *S*. *aureus*. The variations (Table 1) included: muropeptides with 2,6-*N*,*O*-diacetylmuramic acid (NAMOAc); different numbers of Gly residues and substitutions of one or more Gly for Ser or Ala in the bridge peptide; and a number of D-Ala residues. Unexpectedly, the most abundant muropeptide in all instances had a single alanine residue as a bridge peptide (NAG-NAM-A-Q-K(ε-A)-A-A) (Table 1) instead of the canonical poly-Gly (penta)peptide of staphylococcal PG.

**Table 1:** Relative quantification and comparison of the PG muropeptides of *S*. *epidermidis* ST10002 (PIA^+^) and AZ39 (PIA^-^) biofilm cells grown in TSBg and PCs.

Peak Number	Chemical composition	AZ39		Peak Number	Chemical composition	ST10002	
		TSBg	PC			TSBg	PC
		% Corr area	% Corr area			% Corr area	% Corr area
1, 5	NAG-NAM-A-Q-K-A-A	7.05	41.48	1, 5, 6	NAG-NAM-A-Q-K-A-A	9.87	34.48
2	NAG-NAM-A-Q-K-(G)-A-A	5.67	6.52	2	NAG-NAM-A-Q-K-(G)-A-A	5.99	9.57
2	NAG-NAM-A-Q-K-(G2S3)-A-A	0.25	0.00	2	NAG-NAM-A-Q-K-(G2S3)-A-A	0.00	0.00
2	NAG-NAMOAc-A-Q-K-(S3)	1.95	0.96	2	NAG-NAMOAc-A-Q-K-(S3)	0.81	1.41
3	NAG-NAM-A-Q-K-(G4S)-A	0.66	0.00	3	NAG-NAM-A-Q-K-(G-G-G-G-G)-A	2.69	0.11
4	NAG-NAM-A-Q-K-(G-G)-A-A	2.46	1.62	4	NAG-NAM-A-Q-K-(G-G)-A-A	1.49	3.98
4	NAG-NAM-A-Q-K-(G-G-G)-A-A	0.26	0.00	4,5	NAG-NAM-A-Q-K-(G-G-G)-A-A	0.52	0.97
4	NAG-NAM-A-Q-K-(G3S2)-A-A	0.00	1.11	4,6	NAG-NAM-A-Q-K-(G2S)-A-A	0.00	0.84
4,6	NAG-NAM-A-Q-K-(G2S)-A-A	2.25	2.84	4,15	NAG-NAMOAc-A-Q-K-(G3-S2)-A-A	0.63	1.34
5, 6, 7, 20	NAG-NAMOAc-A-Q-K-A-A	4.47	14.07	5	NAG-NAM-A-Q-K-(G-G-G-G)-A-A	0.22	0.25
6	NAG-NAM-A-Q-K-(S3)	1.55	0.00	5, 6, 7, 20	NAG-NAMOAc-A-Q-K-A-A	7.48	4.30
6, 7, 9	NAG-NAM-A-Q-K-(G-G-G-G-G)-A-A	7.16	3.81	6, 7, 9	NAG-NAM-A-Q-K-(G-G-G-G-G)-A-A	5.08	3.20
7	NAG-NAM-A-Q-K-(G-G-S-G-G)-A-A	13.97	3.95	7	NAG-NAM-A-Q-K-(G-G-S-G-G)-A-A	0.00	0.00
8	NAG-NAM-A-Q-K-(A)-A-A	14.84	18.33	8	NAG-NAM-A-Q-K-(A)-A-A	22.5	31.60
11	NAG-NAMOAc-A-Q-K-(G-G-G-G-G)	3.54	1.48	11	NAG-NAMOAc-A-Q-K-(G-G-G-G-G)	8.99	3.73
12	NAG-NAMOAc-A-Q-K-(G-G-G-G-G)-A	0.00	0.71	12	NAG-NAMOAc-A-Q-K-(G-G-G-G-G)-A	0.89	0.95
12	NAG-NAMOAc-A-Q-K-(G4-S)	3.67	0.00	12	NAG-NAMOAc-A-Q-K-(G4-S)	1.27	0.00
12	NAG-NAM-A-Q-K-(G4A)-A-A	2.57	0.18	12	NAG-NAM-A-Q-K-(G4A)-A-A	0.00	0.00
13	NAG-NAM-A-Q-K-(G3AS)-A-A	7.49	0.00	13	NAG-NAM-A-Q-K-(G3AS)-A-A	2.85	0.00
14	NAG-NAMOAc-A-Q-K-(G)-A-A	0.28	0.00	15	NAG-NAMOAc-A-Q-K-(G)-A-A	0.93	0.00
14	NAG-NAMOAc-A-Q-K-(G2-S)-A	0.17	0.00	15	NAG-NAMOAc-A-Q-K-(G2-S)-A	0.55	0.00
14	NAG-NAMOAc-A-Q-K-(G3-S2)-A-A	0.19	0.00	15, 18, 19, 20	NAG-NAMOAc-A-Q-K-(G4-S)-A-A	6.07	0.00
14, 17, 19, 20	NAG-NAMOAc-A-Q-K-(G4-S)-A-A	7.64	0.13	18	NAG-NAMOAc-A-Q-K-(G2S)-A-A	0.00	0.00
17	NAG-NAMOAc-A-Q-K-(G-G)-A-A	0.64	0.00	18	NAG-NAMOAc-A-Q-K-(G-G)-A-A	0.00	0.00
17	NAG-NAMOAc-A-Q-K-(G2S)-A-A	0.40	0.00	19	NAG-NAMOAc-A-Q-K-(G-G-G-G-G)-A-A	0.73	0.00
20	NAG-NAMOAc-A-Q-K-(G-G-G-G-G)-A-A	0.85	0.00	19	NAG-NAMOAc-A-Q-K-(S3)	1.67	0.00
21, 22, 23	NAG-NAMOAc-A-Q-K-(A)-A-A	10.05	2.84	20, 21, 22	NAG-NAMOAc-A-Q-K-(A)-A-A	18.78	3.27
	**TOTAL (%)**	100.02	100.03		**TOTAL (%)**	100.01	100
PG Modifications	**AZ39**	**TSBg**	**PC**	PG Modifications	**ST10002**	**TSBg**	**PC**
	Serine muropeptides	50.30	31.66		Serine muropeptides	13.85	3.59
	O-acetilation	37.48	5.34		O-acetilation	47.99	13.59
	Non A-A muropeptides	38.61	22.41		Non A-A muropeptides	16.87	6.2
	S-link	40.38	26.09		S-link	29.20	9.33

% Corr area = corresponding area under a peak

**Fig 5:**
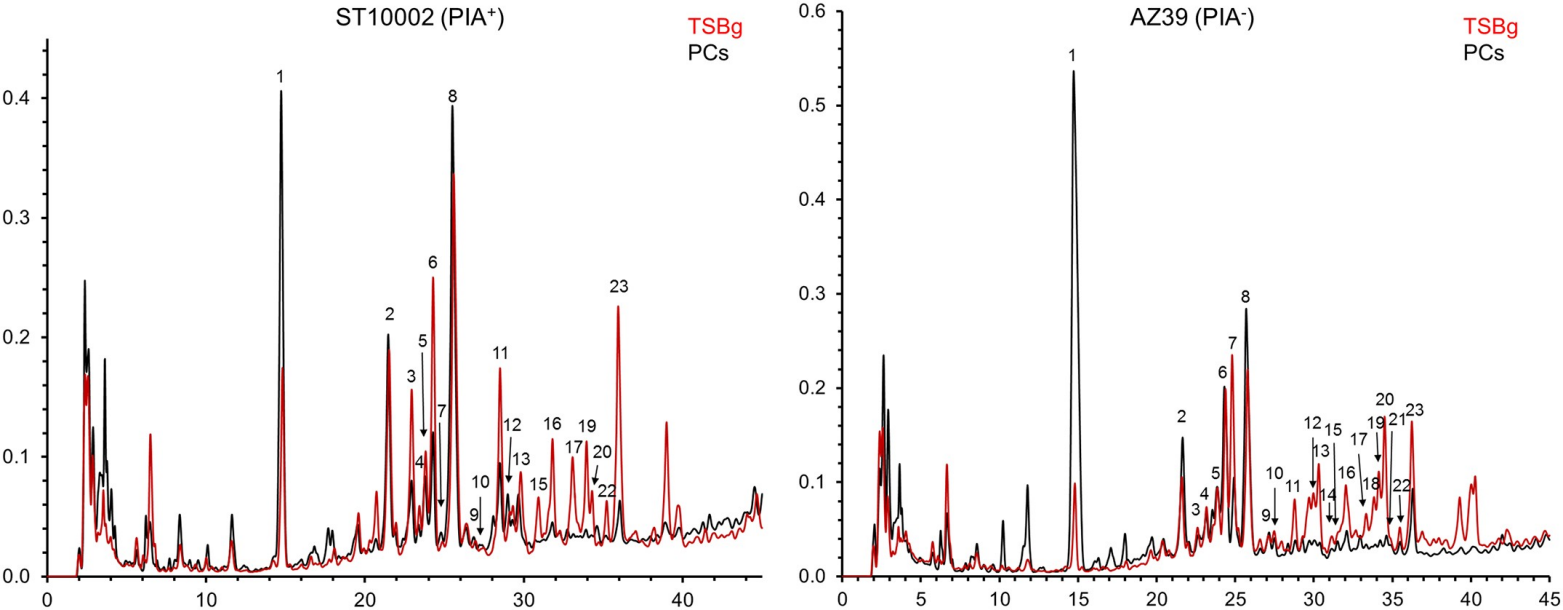
Structural changes in the *S*. *epidermidis* peptidoglycan in PCs. The peptidoglycan profile of *S*. *epidermidis* ST10002 (left) and AZ39 (right) of biofilm cells formed in PCs (black) and in TSBg (red) are overlapped.

Our results indicated a strong influence of the growth environment on the PG composition with similar consequences in both strains. These effects resulted in clearly different HPLC elution patterns (Fig 5 and Fig A in S1 Fig), more complex in the case of biofilms formed in TSBg compared to those formed in PCs. In fact, many of the late eluting components, between Rt 25 to 40 min, were missing in PG from biofilms formed in PCs. Indeed, the proportions of muropeptides with Ser in the bridge peptides, with NAMOAc, with complete (pentapeptidic) bridge peptides, and with D-ala-D-ala as C-terminal dipeptide were all considerably higher in the PG profile from TSBg than from PCs cultures (Table 1).

Furthermore, there was a substantial shift in the nature of the predominant muropeptides from subunits with intact (pentapeptidic) bridge peptides (such as muropeptides with peak numbers 6, 7, 9, 12, 14, 17 and 20) into subunits with a truncated, or missing, bridge-peptide (like muropeptides 1, 2, 8, 12), as shown in Table 1. Comparison of the muropeptide composition for ST10002 (PIA^+^) and AZ39 (PIA^-^) strains grown in TSBg displayed the presence of the same muropeptides but at substantially different proportions. In fact, AZ39 PG had about three times as many Ser-containing muropeptides and twice as many subunits with peptapeptidic bridge-peptides as ST10002 but had a lower degree of O-acetylation (Table 1). Because both strains respond similarly when grown in PCs, the differences between them are maintained, with the exception of O-acetylation, which under these conditions is higher in AZ39 than in ST10002 (Fig A in S1 Fig). Assuming that the results from the analysis of the monomeric subunits is a faithful reflection of total PG, our data support a strong influence of both, environmental conditions and ability to form PIA-based biofilms on PG composition.

## Discussion

In the present work, we have shown that *S*. *epidermidis* biofilm cells undergo structural changes in their PG and biofilm matrix composition when grown in buffy coat PCs compared to optimal laboratory conditions. PCs are known to provide ideal conditions for bacteria to grow; however, the PC milieu also provides a stressful environment with bactericidal properties due to the presence of immune factors such as AMPs [10, 11]. Biofilm formation by *S*. *epidermidis* is likely a survival mechanism to resist such antimicrobial factors [15]. The biofilm has an architecture including the extracellular matrix that provides resistance to antibiotics and other antimicrobial agents in different environments [45]. Here we have shown that the biofilm matrix of different *S*. *epidermidis* strains in PCs is mainly composed of proteins and eDNA, which differs from the chemical structure when the bacteria are grown in regular media. It is important to recognize that proteins associated to the biofilm matrix could also be plasma proteins present in the PCs. These observations open new questions about alternative unexplored mechanisms for biofilm formation that are induced when *S*. *epidermidis* is grown in PCs. Understanding of the proteinaceous nature of the biofilm matrix of *S*. *epidermidis* formed in PCs would provide the identification of potential molecular targets to avoid biofilm formation and consequently improve PC transfusion safety. In *S*. *aureus*, formation of a proteinaceous matrix containing cytoplasmic proteins exported to the cell surface has been described to be linked to a decrease in pH in the growth medium [46]. The molecular mechanisms that regulate this phenomenon remain unclear. The presence of glucose in the growth medium for bacteria leads to the accumulation of acidic by-products from fermentation that is critical for the decrease in pH and the induction of biofilms. In PCs, the high levels of glucose might be one of the factors that contributes to the induction of biofilm formation [47]. It would be reasonable to hypothesize that the utilization of glucose by bacteria could create a microenvironment with a decreased pH that stimulates the exportation of cytoplasmic proteins to the cell surface to form a proteinaceous biofilm matrix. Indeed, a decrease in pH in contaminated PCs has been associated to high titres of bacterial contamination [48, 49].

This is the first study that explored the PG profile of *S*. *epidermidis* biofilms formed in PCs. The muropeptide composition of PG from *S*. *epidermidis*, quite similar to *S*. *aureus* [50], was characterized by the presence of D-Gln, penta-Gly bridges, often with either Ser or Ala substituting for one or more Gly, and muropeptides with O-acetylated NAM. However, *S*. *epidermidis* exhibited a distinct abundance of muropeptides with an Ala substituting for the poly-Gly bridge peptide. The structural changes in the *S*. *epidermidis* cell wall surface might be a strategy to adapt to different environments, in particular to resist clearance by immune factors abundant in PCs [12, 51, 52]. Certainly, the most conspicuous changes observed in our study involved the proportions of muropeptides with NAMOAc, and with Ser residues in the bridge peptides. The presence of Ser residues in the poly-Gly bridge peptide, the absence of a poly-Gly bridge peptide, and the proportion of NAMOAc, modulate resistance of PG against potentially damaging enzymes. In particular, these types of muropeptides have been associated to increased resistance against the PG degrading enzymes lysozyme and lysostaphin [53]. Lysozymes are secreted by neutrophils and macrophages as a defense mechanism. The resistance of *S*. *epidermidis* to lysozymes contribute to its pathogenicity and to its ability to colonize the skin and other human tissues [54]. Surprisingly, the changes detected herein pointed into an unexpected direction. Our results suggest that the PG of *S*. *epidermidis* cells formed in PCs would be more sensitive to degrading enzymes than the PG of biofilm cells grown in TSBg. It is possible that PG hydrolytic enzymes are a relatively minor threat for cells in PCs, which warrants further exploration. The higher proportion of muropeptides with Ala as a bridge peptide in PC-grown biofilms could compensate for the lower Ser content, as absence of penta-Gly also provides lysostaphin resistance. It is therefore postulated that *S*. *epidermidis* minimizes the PG synthesis and synthesizes PG with lower complexity to survive, optimizing energy for other functions required for optimal proliferation in the adverse PC environment. Amidation of D-Glu in PG has a profound impact on the physicochemical properties of the molecules since it removes the negative charge from the D-Glu alpha-carboxyl group [50]. It seems useful as a general mechanism for *S*. *epidermidis*, and other species, to minimize the interaction of PG with cationic AMPs.

Our study was limited to PCs suspended in plasma prepared by the buffy coat method. We recognize that although our work highlights how the PC storage environment induces biofilm matrix and cell wall modification of *S*. *epidermidis*, it is unknown how other manufacturing and/or storage conditions would affect these structural modifications. Although we have previously shown that the presence of platelets [16] and plasma proteins [17, 55] enhance *S*. *epidermidis* biofilm formation, we have not performed comparative biofilm studies between apheresis and buffy coat PCs. We have demonstrated that *S*. *epidermidis* biofilms are resistant to AMPs [15]; however, the resistance mechanisms to other immune factors such as lysozymes present in PCs remain to be investigated. Further studies to address these questions and the potential differences in biofilm matrix and cell wall modification are warranted. Biofilm matrix and cell wall remodeling facilitates bacterial survival in an unhospitable environment. Both features serve *S*. *epidermidis* as mechanisms of defense and although they are commonly studied as two separate systems, here we showed that they are interconnected, as it is the case of other bacteria [56]. This is the first reported study investigating physiological changes undergone by bacteria when grown in PCs. It has generated novel insights that could be used for the development of strategies to mitigate the risk of transfusing bacterially-contaminated PCs, which is one of the greatest post-transfusion infectious risk worldwide [6, 57].

## Supporting information

S1 Fig Comparison of the peptidoglycan profiles of *S*. *epidermidis* ST10002 and AZ39 grown in PCs and in TSBg. (DOCX)

S1 Table Protein detection in *S*. *epidermidis* biofilms. A proteinase K disruption assay shows the presence of a proteinaceous matrix in *S*. *epidermidis* biofilms grown in TSBg and PCs. Mean and standard deviation (SD) were calculated for each strain and growth environment (TSBg or PCs).
(DOCX)

S2 Table eDNA detection in *S*. *epidermidis* biofilms. A DNase I disruption assay shows values indicating the presence of eDNA in the matrix of *S*. *epidermidis* biofilms grown in TSBg. The difference between untreated and DNase-treated biofilms grown in TSBg is significant for all the tested strains (p<0.02) except for 9142 *ΔicaA* (p = 0.0775). No significant difference (p>0.05) was found between untreated and DNAse-treated biofilms formed in PCss.
(DOCX)

S3 Table CLSM signal intensities. For visualization of WGA-OG488, SYPRO-Ruby and DAPI staining, excitation lasers of 488 nm, 405 nm and 405 nm were used, respectively. For the TSBg samples, emission bands of 505–555 nm for WGA-OG488, 555–700 nm for SYPRO-Ruby, and 410–505 nm for DAPI were acquired using the GaAsP detectors and a Plan-Apochromat 40x NA 1.4 oil objective. For the PC samples, emission bands of 485–560 nm for WGA-OG488, 554–700 nm for SYPRO-Ruby, and 400–530 nm for DAPI were acquired using the high resolution Airyscan detector and a Plan-Apochromat 63x NA 1.4 oil objective.
(DOCX)

S4 Table CLSM signal intensities. Samples were visualized using excitation lasers of 488 nm and 640 nm for TOTO-1 and SYTO-60, respectively. An emission band of 450–630 nm was acquired for TOTO-1 using the Airyscan detector and an emission band of 656–700 nm was acquired for SYTO-60 using the GaAsP detector. Differential interference contrast microscopy (DIC) was used to observe the biofilms without stain.
(DOCX)
