# Supplementary material for: The peptidoglycan and biofilm matrix of Staphylococcus epidermidis undergo structural changes when exposed to human platelets
Source: PLoS One. 2019 Jan 25;14(1):e0211132. doi: 10.1371/journal.pone.0211132 (PMC6347161; doi:10.1371/journal.pone.0211132)
Supplement: S1 Fig — (DOCX) [file pone.0211132.s001.docx]

**The peptidoglycan and biofilm matrix of *Staphylococcus epidermidis* undergo structural changes when exposed to human platelets**

Maria Loza-Correa^1,2^, Juan A Ayala^3^, Iris Perelman^1^, Keith Hubbard^4^, Miloslav Kalab^4^, Qi-Long Yi^1^, Mariam Taha^1^, Miguel A. de Pedro^3^, and Sandra Ramirez-Arcos^1,2*^

^1^Centre for Innovation, Canadian Blood Services, Ottawa, Canada

^2^Department of Biochemistry, Microbiology and Immunology, University of Ottawa, Ottawa, Canada

^3^Centro de Biología Molecular Severo Ochoa, Universidad Autónoma de Madrid, Madrid, Spain

^4^Agriculture and Agri-food Canada, Ottawa, ON, Canada


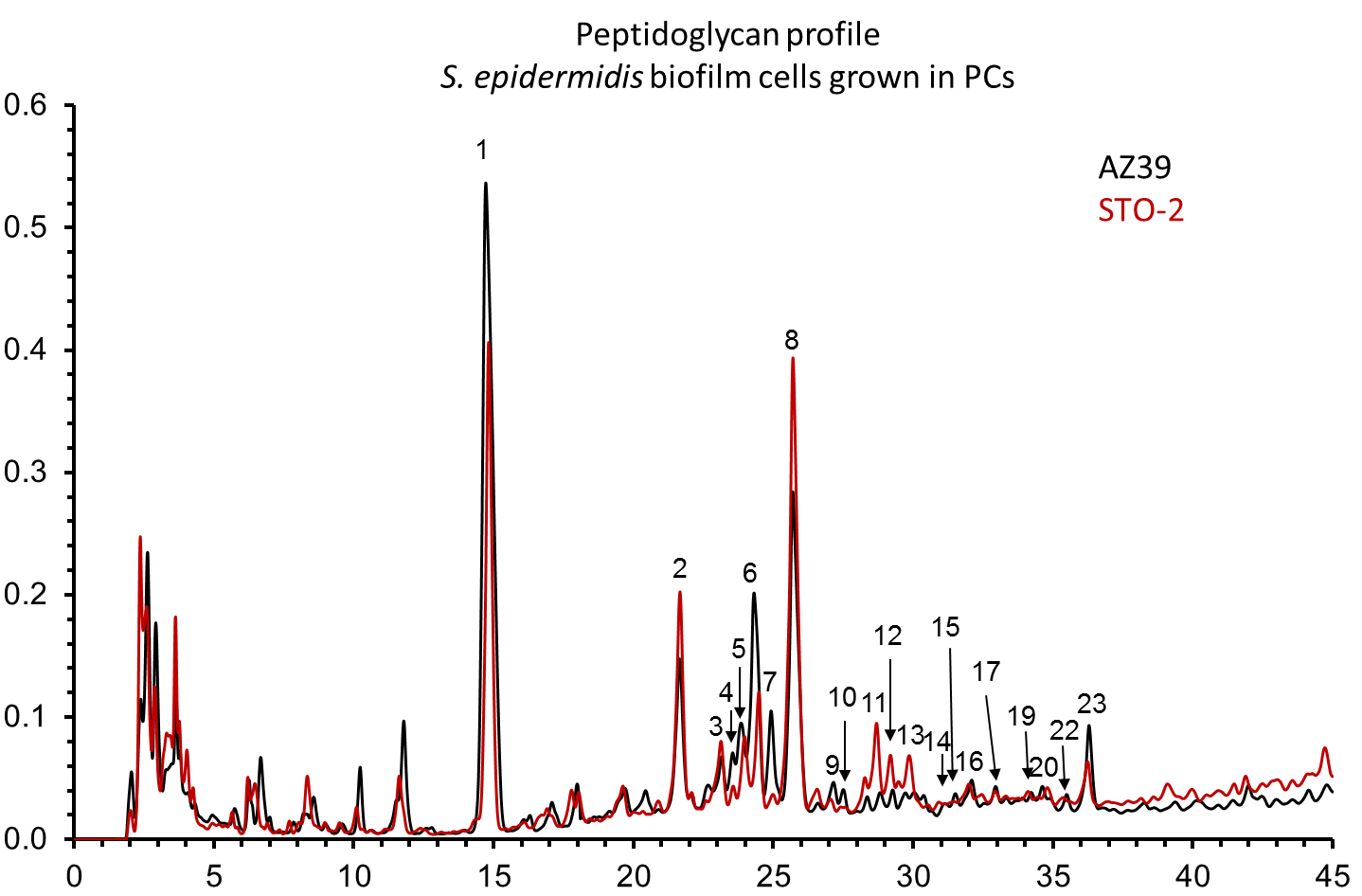

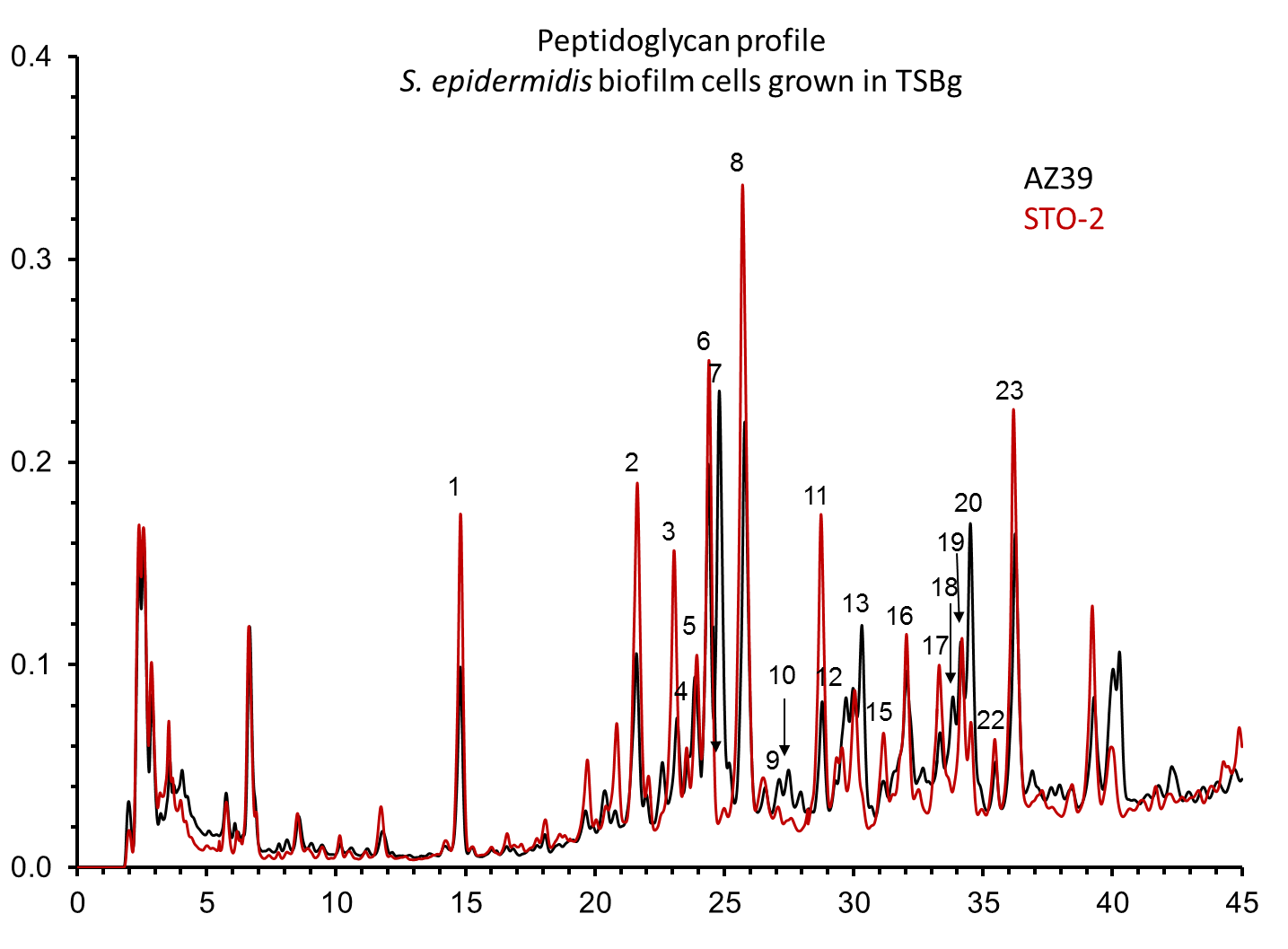


**S1 Figure. Comparison of the peptidoglycan profiles of *S. epidermidis* ST10002 and AZ39 grown in PCs and in TSBg.**
