# Supplementary material for: The peptidoglycan and biofilm matrix of Staphylococcus epidermidis undergo structural changes when exposed to human platelets
Source: PLoS One. 2019 Jan 25;14(1):e0211132. doi: 10.1371/journal.pone.0211132 (PMC6347161; doi:10.1371/journal.pone.0211132)
Supplement: S1 Table — A proteinase K disruption assay shows the presence of a proteinaceous matrix in S. epidermidis biofilms grown in TSBg and PCs. Mean and standard deviation (SD) were calculated for each strain and growth environment (TSBg or PCs). (DOCX) [file pone.0211132.s002.docx]

**The peptidoglycan and biofilm matrix of *Staphylococcus epidermidis* undergo structural changes when exposed to human platelets**

Maria Loza-Correa^1,2^, Juan A Ayala^3^, Iris Perelman^1^, Keith Hubbard^4^, Miloslav Kalab^4^, Qi-Long Yi^1^, Mariam Taha^1^, Miguel A. de Pedro^3^, and Sandra Ramirez-Arcos^1,2*^

^1^Centre for Innovation, Canadian Blood Services, Ottawa, Canada

^2^Department of Biochemistry, Microbiology and Immunology, University of Ottawa, Ottawa, Canada

^3^Centro de Biología Molecular Severo Ochoa, Universidad Autónoma de Madrid, Madrid, Spain

^4^Agriculture and Agri-food Canada, Ottawa, ON, Canada

**S1 Table. Protein detection in *S. epidermidis* biofilms**

| **Growth Environment** | ***S. epidermidis* strain** | **Experiment Repetition Number** | **O.D. 492 nm** |  | **Mean (SD)** |  | **p-value** |
| --- | --- | --- | --- | --- | --- | --- | --- |
|  |  |  | **Untreated biofilm** | **Proteinase K** | **Untreated biofilm** | **Proteinase K** |  |
|  | 9142 | 1 | 0.163 | 0.168 | 0.20 | 0.17 | 0.1206 |
|  |  | 2 | 0.189 | 0.146 | (0.02) | 0.02) |  |
|  |  | 3 | 0.213 | 0.195 |  |  |  |
|  |  | 4 | 0.216 | 0.196 |  |  |  |
|  | 9142ΔicaA | 1 | 0.084 | 0.045 | 0.10 | 0.05 | 0.0098 |
|  |  | 2 | 0.120 | 0.051 | (0.02) | (0.01) |  |
|  |  | 3 | 0.068 | 0.036 |  |  |  |
|  |  | 4 | 0.112 | 0.062 |  |  |  |
| TSBg | AZ-22 | 1 | 0.334 | 0.061 | 0.42 | 0.06 | 0.0229 |
|  |  | 2 | 0.283 | 0.049 | (0.18) | (0.01) |  |
|  |  | 3 | 0.372 | 0.053 |  |  |  |
|  |  | 4 | 0.675 | 0.077 |  |  |  |
|  | AZ-39 | 1 | 0.700 | 0.025 | 0.80 | 0.03 | 0.0044 |
|  |  | 2 | 0.793 | 0.038 | (011) | (0.01) |  |
|  |  | 3 | 1.244 | 0.035 |  |  |  |
|  | ST10002 | 1 | 0.250 | 0.190 | 0.23 | 0.17 | 0.0301 |
|  |  | 2 | 0.267 | 0.191 | (0.05) | (0.03) |  |
|  |  | 3 | 0.179 | 0.139 |  |  |  |
|  | 9142 | 1 | 0.766 | 0.074 | 0.76 | 0.05 | 0.0015 |
|  |  | 2 | 0.822 | 0.061 | (0.07) | (0.03) |  |
|  |  | 3 | 0.684 | 0.013 |  |  |  |
|  | 9142ΔicaA | 1 | 0.534 | 0.043 | 0.53 | 0.02 | 0.0027 |
|  |  | 2 | 0.484 | 0.021 | (0.04) | (0.02) |  |
|  |  | 3 | 0.567 | 0.005 |  |  |  |
|  | AZ-22 | 1 | 0.067 | 0.021 | 0.13 | 0.01 | 0.0685 |
| PCs |  | 2 | 0.144 | 0.008 | (0.06) | (0.01) |  |
|  |  | 3 | 0.174 | 0.006 |  |  |  |
|  | AZ-39 | 1 | 1.054 | 0.000 | 0.59 | 0.00 | 0.1667 |
|  |  | 2 | 0.098 | 0.002 | (0.48) | (0.00) |  |
|  |  | 3 | 0.625 | 0.007 |  |  |  |
|  | ST10002 | 1 | 0.168 | 0.197 | 0.30 | 0.08 | 0.261 |
|  |  | 2 | 0.169 | 0.020 | (0.22) | (0.10) |  |
|  |  | 3 | 0.550 | 0.016 |  |  |  |
|  | ST10003 | 1 | 0.162 | 0.036 | 0.26 | 0.02 | 0.117 |
|  |  | 2 | 0.177 | 0.024 | (0.15) | (0.01) |  |
|  |  | 3 | 0.431 | 0.011 |  |  |  |
