# Supplementary material for: The peptidoglycan and biofilm matrix of Staphylococcus epidermidis undergo structural changes when exposed to human platelets
Source: PLoS One. 2019 Jan 25;14(1):e0211132. doi: 10.1371/journal.pone.0211132 (PMC6347161; doi:10.1371/journal.pone.0211132)
Supplement: S2 Table — A DNase I disruption assay shows values indicating the presence of eDNA in the matrix of S. epidermidis biofilms grown in TSBg. The difference between untreated and DNase-treated biofilms grown in TSBg is significant for all the tested strains (p<0.02) except for 9142 ΔicaA (p = 0.0775). No significant difference (p>0.05) was found between untreated and DNAse-treated biofilms formed in PCss. (DOCX) [file pone.0211132.s003.docx]

**The peptidoglycan and biofilm matrix of *Staphylococcus epidermidis* undergo structural changes when exposed to human platelets**

Maria Loza-Correa^1,2^, Juan A Ayala^3^, Iris Perelman^1^, Keith Hubbard^4^, Miloslav Kalab^4^, Qi-Long Yi^1^, Mariam Taha^1^, Miguel A. de Pedro^3^, and Sandra Ramirez-Arcos^1,2*^

^1^Centre for Innovation, Canadian Blood Services, Ottawa, Canada

^2^Department of Biochemistry, Microbiology and Immunology, University of Ottawa, Ottawa, Canada

^3^Centro de Biología Molecular Severo Ochoa, Universidad Autónoma de Madrid, Madrid, Spain

^4^Agriculture and Agri-food Canada, Ottawa, ON, Canada

**S2 Table. eDNA detection in *S. epidermidis* biofilms**.

| **Growth Environment** | ***S. epidermidis* strain** | **Experiment Repetition Number** | **O.D. 492 nm** |  | **Mean (SD)** |  | **p-value** |
| --- | --- | --- | --- | --- | --- | --- | --- |
|  |  |  | **Untreated biofilm** | **DNase I** | **Untreated biofilm** | **DNase I** |  |
|  | 9142 | 1 | 0.189 | 0.111 | 0.22 | 0.13 | 0.022 |
|  |  | 2 | 0.218 | 0.135 | (0.04) | (0.02) |  |
|  |  | 3 | 0.263 | 0.140 |  |  |  |
|  | 9142ΔicaA | 1 | 0.108 | 0.025 | 0.08 | 0.02 | 0.078 |
|  |  | 2 | 0.078 | 0.024 | (0.03) | (0.01) |  |
|  |  | 3 | 0.040 | 0.013 |  |  |  |
| TSBg | AZ-22 | 1 | 1.237 | 0.053 | 1.29 | 0.05 | 0.021 |
|  |  | 2 | 1.631 | 0.066 | (0.32) | (0.01) |  |
|  |  | 3 | 0.988 | 0.040 |  |  |  |
|  | AZ-39 | 1 | 0.262 | 0.030 | 0.26 | 0.03 | 0.008 |
|  |  | 2 | 0.220 | 0.022 | (0.04) | (0.00) |  |
|  |  | 3 | 0.294 | 0.025 |  |  |  |
|  | ST10002 | 1 | 0.170 | 0.024 | 0.0172 | 0.019 | 0.0004 |
|  |  | 2 | 0.174 | 0.015 | (0.002) | (0.005) |  |
|  |  | 3 | 0.172 | 0.019 |  |  |  |
|  | 9142 | 1 | 0.440 | 0.503 | 0.30 | 0.48 | 0.092 |
|  |  | 2 | 0.196 | 0.436 | (0.13) | (0.04) |  |
|  |  | 3 | 0.269 | 0.511 |  |  |  |
|  | 9142ΔicaA | 1 | 0.367 | 0.269 | 0.20 | 0.30 | 0.466 |
|  |  | 2 | 0.075 | 0.180 | (0.15) | (0.14) |  |
|  |  | 3 | 0.156 | 0.459 |  |  |  |
|  | AZ-22 | 1 | 0.193 | 0.184 | 0.16 | 0.18 | 0.831 |
| PCs |  | 2 | 0.096 | 0.249 | (0.06) | (0.07) |  |
|  |  | 3 | 0.204 | 0.100 |  |  |  |
|  | AZ-39 | 1 | 0.147 | 0.149 | 0.34 | 0.36 | 0.854 |
|  |  | 2 | 0.241 | 0.461 | (0.25) | (0.18) |  |
|  |  | 3 | 0.624 | 0.470 |  |  |  |
|  | ST10002 | 1 | 0.228 | 0.167 | 0.30 | 0.31 | 0.251 |
|  |  | 2 | 0.388 | 0.465 | (0.09) | (0.15) |  |
|  |  | 3 | 0.249 | 0.288 |  |  |  |
|  | ST10003 | 1 | 0.228 | 0.167 | 0.23 | 0.23 | 0.377 |
|  |  | 2 | 0.102 | 0.155 | (0.16) | (0.18) |  |
|  |  | 3 | 0.368 | 0.423 |  |  |  |
