# Supplementary material for: The peptidoglycan and biofilm matrix of Staphylococcus epidermidis undergo structural changes when exposed to human platelets
Source: PLoS One. 2019 Jan 25;14(1):e0211132. doi: 10.1371/journal.pone.0211132 (PMC6347161; doi:10.1371/journal.pone.0211132)
Supplement: S3 Table — For visualization of WGA-OG488, SYPRO-Ruby and DAPI staining, excitation lasers of 488 nm, 405 nm and 405 nm were used, respectively. For the TSBg samples, emission bands of 505–555 nm for WGA-OG488, 555–700 nm for SYPRO-Ruby, and 410–505 nm for DAPI were acquired using the GaAsP detectors and a Plan-Apochromat 40x NA 1.4 oil objective. For the PC samples, emission bands of 485–560 nm for WGA-OG488, 554–700 nm for SYPRO-Ruby, and 400–530 nm for DAPI were acquired using the high resolution Airyscan detector and a Plan-Apochromat 63x NA 1.4 oil objective. (DOCX) [file pone.0211132.s004.docx]

**The peptidoglycan and biofilm matrix of *Staphylococcus epidermidis* undergo structural changes when exposed to human platelets**

Maria Loza-Correa^1,2^, Juan A Ayala^3^, Iris Perelman^1^, Keith Hubbard^4^, Miloslav Kalab^4^, Qi-Long Yi^1^, Mariam Taha^1^, Miguel A. de Pedro^3^, and Sandra Ramirez-Arcos^1,2*^

^1^Centre for Innovation, Canadian Blood Services, Ottawa, Canada

^2^Department of Biochemistry, Microbiology and Immunology, University of Ottawa, Ottawa, Canada

^3^Centro de Biología Molecular Severo Ochoa, Universidad Autónoma de Madrid, Madrid, Spain

^4^Agriculture and Agri-food Canada, Ottawa, ON, Canada

**S3 Table. CLSM signal intensities**.

|  | **Arithmetic Mean Intensity** | | | | | |
| --- | --- | --- | --- | --- | --- | --- |
|  | **TSBg** | | | **PCs** | | |
|  | **ST10002** | **AZ39** | **Control** | **ST10002** | **AZ39** | **Control** |
| WGA-OG488 | 3017.16 | 2073.87 | 344.38 | 1006.67 | 589.53 | 137.68 |
| SYPRO-Ruby | 12780.85 | 9912.85 | 1285.97 | 434.55 | 277.99 | 63.26 |
| DAPI | 9530.84 | 5244.78 | 2629.17 | 1201.10 | 521.79 | 20.68 |
