# Supplementary material for: The peptidoglycan and biofilm matrix of Staphylococcus epidermidis undergo structural changes when exposed to human platelets
Source: PLoS One. 2019 Jan 25;14(1):e0211132. doi: 10.1371/journal.pone.0211132 (PMC6347161; doi:10.1371/journal.pone.0211132)
Supplement: S4 Table — Samples were visualized using excitation lasers of 488 nm and 640 nm for TOTO-1 and SYTO-60, respectively. An emission band of 450–630 nm was acquired for TOTO-1 using the Airyscan detector and an emission band of 656–700 nm was acquired for SYTO-60 using the GaAsP detector. Differential interference contrast microscopy (DIC) was used to observe the biofilms without stain. (DOCX) [file pone.0211132.s005.docx]

**The peptidoglycan and biofilm matrix of *Staphylococcus epidermidis* undergo structural changes when exposed to human platelets**

Maria Loza-Correa^1,2^, Juan A Ayala^3^, Iris Perelman^1^, Keith Hubbard^4^, Miloslav Kalab^4^, Qi-Long Yi^1^, Mariam Taha^1^, Miguel A. de Pedro^3^, and Sandra Ramirez-Arcos^1,2*^

^1^Centre for Innovation, Canadian Blood Services, Ottawa, Canada

^2^Department of Biochemistry, Microbiology and Immunology, University of Ottawa, Ottawa, Canada

^3^Centro de Biología Molecular Severo Ochoa, Universidad Autónoma de Madrid, Madrid, Spain

^4^Agriculture and Agri-food Canada, Ottawa, ON, Canada

**S4 Table. CLSM signal intensities**.

|  | **Arithmetic Mean Intenisty** | | |
| --- | --- | --- | --- |
|  | **PCs** | | |
|  | **ST10002** | **AZ39** | **Control** |
| TOTO-1 | 22494.43 | 25655.29 | 303.74 |
| SYTO-60 | 6580.10 | 6932.62 | 287.24 |
| DIC | 38979.79 | 40491.66 | 29260.12 |
